# Increased nuchal translucency in children with congenital heart defects and normal karyotype—is there a correlation with mortality?

**DOI:** 10.3389/fped.2023.1104179

**Published:** 2023-02-17

**Authors:** Rasmus Kristensen, Camilla Omann, J. William Gaynor, Line Rode, Charlotte K. Ekelund, Vibeke E. Hjortdal

**Affiliations:** ^1^ Department of Cardiothoracic Surgery, Copenhagen University Hospital—Rigshospitalet, Copenhagen, Denmark; ^2^ Department of Clinical Medicine, Aarhus University, Aarhus, Denmark; ^3^ Department of Cardiothoracic & Vascular Surgery, Aarhus University Hospital, Skejby, Denmark; ^4^ Division of Cardiothoracic Surgery, Children's Hospital of Philadelphia, Philadelphia, PA, United States; ^5^ Department of Obstetrics, Center for Fetal Medicine, Copenhagen University Hospital—Rigshospitalet, Copenhagen, Denmark; ^6^ Department of Clinical Biochemistry, Copenhagen University Hospital—Rigshospitalet Glostrup, Glostrup, Denmark

**Keywords:** congenital heart defects, nuchal translucency, mortality, prenatal screening, ultrasound, fetal medicine

## Abstract

**Objectives:**

Our objective was to investigate if an increased nuchal translucency (NT) was associated with higher mortality in chromosomally normal children with congenital heart defects (CHD).

**Methods:**

In a nationwide cohort using population-based registers, we identified 5,633 liveborn children in Denmark with a pre- or postnatal diagnosis of CHD from 2008 to 2018 (incidence of CHD 0.7%). Children with chromosomal abnormalities and non-singletons were excluded. The final cohort compromised 4,469 children. An increased NT was defined as NT > 95th-centile. Children with a NT > 95th-centile vs. NT < 95th-centile including subgroups of simple- and complex CHD were compared. Mortality was defined as death from natural causes, and mortalities were compared among groups. Survival analysis with Cox-regression was used to compare rates of mortality. Analyses were adjusted for mediators (possibly explanatory factors between increased NT and higher mortality): preeclampsia, preterm birth and small for gestational age. And for confounding effects of extracardiac anomalies and cardiac intervention, due to their close association to both the exposure and the outcome (i.e., confounders).

**Results:**

Of the 4,469 children with CHD, 754 (17%) had complex CHD and 3,715 (83%) simple CHD. In the combined group of CHDs the mortality rate was not increased when comparing those with a NT > 95th-centile to those with a NT < 95th-centile [Hazard ratio (HR) 1.6, 95%CI 0.8;3.4, *p* = 0.2]. In simple CHD there was a significantly higher mortality rate with a HR of 3.2 (95%CI: 1.1;9.2, *p* = 0.03) when having a NT > 95th centile. Complex CHD had no differences in mortality rate between a NT > 95th-centile and NT < 95th-centile (HR 1.1, 95%CI: 0.4;3.2, *p* = 0.8). All analysis adjusted for severity of CHD, cardiac operation and extracardiac anomalies. Due to limited numbers the association to mortality for a NT > 99th centile (>3.5 mm) could not be assessed. Adjustment for mediating (preeclampsia, preterm birth, small for gestational age) and confounding variables (extracardiac anomalies, cardiac intervention) did not alter the associations significantly, except for extracardiac anomalies in simple CHD.

**Conclusion:**

An increased NT > 95th-centile is correlated with higher mortality in children with simple CHD, but the underlying cause is unknown and undetected abnormal genetics might explain the correlation rather than the increased NT itself, hence further research is warranted.

## Introduction

Factors of an impaired fetal environment have mostly related to the maternal- or placental side of the fetus. The use of ultrasound in prenatal screening has made it possible to investigate the fetal environment on the fetus itself, including anatomical details such as the nuchal translucency.

The Nuchal Translucency (NT) is measured routinely in Denmark as part of the first trimester combined prenatal-screening, due to its association with abnormal fetal karyotype (1–3). An increased NT is also associated with increased risk of congenital heart defects (CHD) (4–9), other structural abnormalities and rare syndromes (10–14). In addition, an increased NT is associated with adverse pregnancy outcomes such as spontaneous abortion and fetal death (14). These risks and associations apply to euploid fetuses as well.

The pathogenesis of an increased NT is a topic of debate, with the main theories centering around the causes being abnormal development of lymphatic vessels, extracellular matrix changes and cardiac abnormalities (1, 4, 15–17).

Whether malformation of the lymphatics causes an increased NT and CHD or whether CHD causes altered fluid-flows, lymphatics and then an increased NT is unknown. The association is most likely multifactorial and causality is difficult to determine (8, 15–18). The cardiovascular system and the lymphatics are intertwined and perhaps even more in CHD, where lymphatic abnormalities are associated with several comorbidities (19, 20).

Until now no studies have investigated the NTs possible role as a proxy of an impaired fetal environment in children with CHD and how this may affect their postnatal mortality. We hypothesize that an increased NT is associated with higher postnatal mortality in children with CHD. Our aim was to investigate the association between increased NT and postnatal mortality in children with CHD.

## Materials and methods

### Study design and data sources

We designed a nationwide cohort study including all liveborn children with CHD in Denmark from 2008 to 2018, using the following registers: The Danish National Patient Register (21, 22), with data on all hospital admissions and diagnoses; The Danish Medical Birth Register, with data on all births in Denmark and linkage of child and mother (23, 24); The Danish Cytogenetic Central Regiser (25), with data on all cytogenetic tests in Denmark.

Since 1968 all Danes have been provided with a unique personal identification number that allows for linkage across these national registers (21, 22, 24).

As part of access to tax-funded public free healthcare in Denmark, pregnant women are offered a first trimester combined screening and a second trimester scan for fetal anomalies. More than 90% of all pregnant Danish women participate in the screening program (2, 26). Our cohort was matched with the Danish Fetal Medicine Database which holds prenatal screening information including the NT measurements (2, 27).

### Study population

We included all liveborn children between 2008 and 2018 with a diagnosis of CHD. Only patients with a diagnosis given at a university hospital were included, to increase validity, similar to methods used in previous studies (28–30).

**Figure 1:**
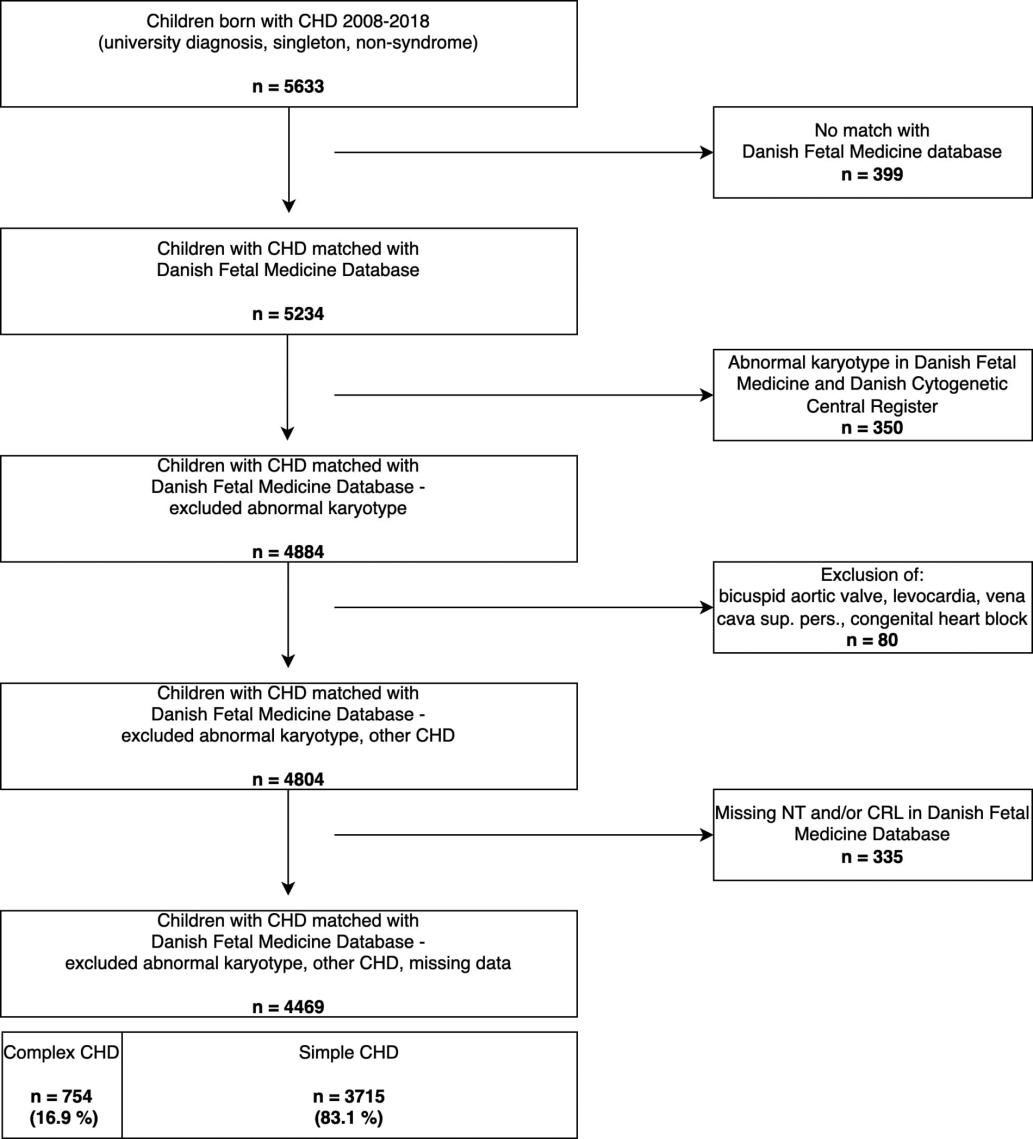
Participants flow.

Children with chromosomal anomalies: trisomy 21, 13, 18; DiGeorge-syndrome; Turner-syndrome; William-Beuren; Klinefelter; or with a genetic analysis marked “abnormal karyotype” were excluded, due to the confounding association of genetic syndromes with both the NT and excess mortality and its strong association to CHD (1, 31), as in similar previous studies (28, 32, 33). The methods for prenatal detection of genetic anomalies at the genetic departments and the Danish Cytogenetic Central Register transitioned from conventional karyotyping to chromosomal microarray gradually over the course of the study period. Data on copy-number-variants and RASopathies were not available in our dataset. From 2013 all children with a NT ≥ 99th centile (3.5 mm) were tested with chromosomal microarray independent of their first-trimester risk-assessment. Postnatal detection of genetic anomalies involved chromosomal microarray for the entire study period. Chromosomal anomalies were excluded in two steps as these were identified from two different registers (depicted in Figure 1).

### Categorization of congenital heart defects

CHD was defined as ICD-10 codes in the Danish National Patient Registry: DQ20—DQ26, including sub-codes, and categorized into either “simple” or “complex” CHD, based on a modification of the 2018 American Heart Association guidelines (34) and previous studies (30, 33).

The specific CHD of each patient was identified from the primary diagnosis of each university hospital contact. If more than one CHD per contact or patient, the most severe defect given at the earliest point in time was chosen similar to previous studies and hierarchy (30, 33–38).

For the ICD-10 codes included in each subtype of CHD see Supplementary Table S1A. All subtypes of CHD within “simple” and “complex” CHD are shown in Table 2, and hierarchically arranged. Unspecific codes for CHD, and codes for nonstructural CHD were not included (ICD-10; DQ231A, DQ241, DQ246, DQ261).

### Nuchal translucency

The NT is routinely measured in gestational week 11 to 13 + 6, as part of the first trimester prenatal-screening. The Danish sonographers adhere to the protocol of the Fetal Medicine Foundation (3) for scanning the NT. The first-trimester prenatal screening for syndromes and congenital anomalies include; Double-test with blood tests for PAPP-A and beta-hCG in gestational week 8–14, NT-measurement in gestational week 11–14. A risk-score is calculated based on the values from the double-test and the nuchal translucency and the maternal age. If the risk is above 1:300 for trisomy 21 and above 1:150 for trisomy 18 and 13, further diagnostics are offered. These further diagnostics include chorionic villus sampling with chromosomal microarray/array-CGH or amniocentesis. Non-invasive prenatal testing can be offered as an alternative to the further diagnostics, but this is not implemented as a routine or stand-alone tool by the Danish Fetal Medicine Society. In second trimester, gestational week 20–22, pregnant women are also offered a free fetal ultrasound scan to detect any fetal malformations. Prenatal screening is offered to all pregnant women. The screening is free-of-charge as part of access to tax-funded public free healthcare and >90% of all pregnant women attend this.

The NT was divided into NT < 95th centile or NT ≥ 95th centile. This cut-off was chosen as the 95th centile denotes an “increased” nuchal translucency, and a NT above the 95th warrants further prenatal testing. The 99th centile (3.5 mm) was included in the NT ≥ 95th centile, as this contained too few patients to analyze. The NT centiles were calculated based on the crown-rump-length (CRL) at the first trimester scan using the method and model as by the Fetal Medicine Foundation (39).

### Outcomes

The primary outcomes were mortality (numbers) and mortality rates (deaths per unit of time) for children with any type of CHD, simple CHD and complex CHD.

Mortality was identified in the Danish Register of Causes of Death (40). Only death from “natural causes” (i.e., from illness) were included; death from accidents, suicide or violence were excluded (40).

### Covariates

Pre-eclampsia (PE) was defined as ICD-10 codes D014-D0149, in line with previous studies using preeclampsia (41).

Preterm birth (PTB) was defined as birth before gestational week 37.

Small for gestational age (SGA) was defined as birthweight below the 10th-percentile for the gestational age (equivalent to −15% of expected birthweight for the given gestational age derived from Maršál et al. (42)).

PE, PTB, SGA were all considered as potential mediators between increased NT and higher mortality.

Extracardiac anomalies were defined as having any other congenital extracardiac anomaly and identified in the Danish National Patient Registry by ICD-10 codes; DQ00–07, DQ10–18, DQ30–34, DQ35–37, DQ38–45, DQ50–56, DQ60–64, DQ65–79, DQ80–89. Extracardiac anomalies were considered a potential confounder.

Operation, i.e., undergoing cardiac intervention, was pooled into a single dichotomized (yes/no) covariate. Patients undergoing any type of cardiac operation at any point in time were identified from the Danish National Patient Register where operations are classified and coded according to the Nordic Medico-Statistical Classification of Surgical Procedures (43). If a patient had more than one operation, the operation with the highest risk according to RACHS-1 category was chosen (44). Undergoing operation was adjusted for in the final model due to its close association to mortality and *via* the type of CHD to nuchal translucency.

Confounders are associated with both the exposure and outcome, whereas mediators are intermediate possibly explanatory factors between the exposure and outcome.

### Statistical analysis

Baseline characteristics were reported as mean with standard deviation for normally distributed variables, and as interquartile intervals or range for not normally distributed data. Normally distributed variables were compared using Students t-test and not normally distributed using Kruskal-Wallis test.

Categorical variables were reported in percentages and compared using chi-squared test and fishers exact test when appropriate.

For the primary analysis of a NT ≥95th–vs. <95th centile on the outcome of mortality, Kaplan-Meier survival curves and Cox-regression were used to compare mortality rates and adjust for covariates. The lack of complete follow-up for all patients were accounted for by using time-to-event analysis i.e., Cox-regression.

Survival time in the Cox-regression was time from birth until either death or end-date of study (31st of December 2019). If there was no record of death the children were presumed to be alive. The Danish National Register on Death provided information on death on our patients until 31st of December 2019, hence this was chosen as end-date of the study.

The analyses were adjusted for confounding and mediation by covariates in the Cox-regression.

PE, PTB, SGA were introduced in the model as mediators. The type of CHD (i.e., simple or complex), undergoing any cardiac operation and extracardiac anomalies were introduced as confounding variables. The confounding by “chromosomal anomalies” was addressed by excluding these. The goal by adjusting for these variables were to understand the direct relationship between the exposure, the NT, and the outcome, mortality.

The level of statistical significance was set at 0.05. Stata SE version 15.0 (Stata-Corp, College Station, Texas) on Statistics Denmark's encrypted online data service was used for analyses.

### Approvals

The study was approved by the regional data protection agency (approval number: P-2020-183). As per the Danish Data Protection Law §10, informed consent is not needed for Danish registry-based research studies.

## Results

### Participants and participant flow

We identified 5,633 children with CHD of the 663,616 liveborn children from 2008 to 2018 from the Danish National Birth- and The Danish National Patient Register (Figure 1).

We matched these with the Danish Fetal Medicine Database. A further 1,164 children were excluded due to non-matching, additional information on abnormal karyotype stated as “other abnormal karyotype” in the Danish Fetal Medicine Database, non-structural CHD, missing information on the NT or CRL (Figure 1, steps 1–4). The final study cohort included 4,469 liveborn children with CHD and a NT measurement.

### Baseline characteristics

Our study population comprised 4,469 children with CHD; 3,715 with simple and 754 with complex (Table 1).

**Table 1:** Study Population characteristics.

Variables	*n*
All patients 2008–2018 (‰ of live births)	4,469 (6.7)
**Maternal health**	
Maternal age at NT-scan (years), mean (sd)	29.8 (5.0)
Maternal BMI WHO-category, *n* (%)	4,361 (100)
– Underweight	292 (6.7)
– Normal	2,413 (55.3)
– Overweight	978 (22.4)
Obese	678 (15.5)
**Patient characteristics**	
Sex (female), *n* (%)	2,159 (48.3)
**CHD characteristic *n* (%)**	
All type CHD	4,469 (100)
Simple CHD	3,715 (83.1)
Complex CHD	754 (16.9)
**First trimester screening**	
Nuchal translucency (mm.), median (iqi)	1.7 (1.4;2.0)
Nuchal translucency ≥ 3.5 mm. (yes), *n* (%)	57 (1.3)
GA at NT-scan (weeks), mean (sd)	12 (1)
**Birth variables**	
•GA at birth (days), median (iqr)	276 (18)
•Birthweight (g.), mean (sd)	3,205 (840)

CHD, congenital heart defect; GA, gestational age; NT, nuchal translucency; iqi, interquartile interval.

The children born with CHD had a mean gestational age at birth of 269 days, and a mean birthweight of 3,205 gram (Table 1). The mean maternal age was 29.8 years at childbirth and the mothers went to the first trimester NT-scan at a mean GA of the fetus of 12 weeks. The distribution of subtypes of CHD within simple and complex CHD and if the NT was ≥95th centile is seen in Table 2.

**Table 2:** Distribution of subtypes within simple and complex CHD and a NT ≥ 95th centile.

CHD and subtype	*n* (%)	NT ≥ 95th Centile, *n* (%)
All CHD	4469	216 (4.8)
Complex CHD	754 (16.9)	62 (8.2)
– AVSD	262 (5.9)	13 (5.0)
– TGA	169 (3.8)	15 (8.9)
– TOF	94 (2.1)	10 (10.6)
– Complex miscellaneous	88 (2.0)	5 (5.7)
– I/HAA	31 (0.7)	5 (16.1)
– Non-HLHS single ventricle	20 (0.4)	5 (25.0)
– HLHS	20 (0.4)	<5 (.)
– TAPVD	20 (0.4)	<5 (.)
– Ebsteins Anomaly	18 (0.4)	<5 (.)
– Tricuspid Valve Disease	12 (0.3)	<5 (.)
– Pulmonary atresia	12 (0.3)	0 (0.0)
– Common Arterial Trunk	8 (0.2)	0 (0.0)
Simple CHD	3,715 (83.1)	154 (4.1)
– VSD	1,294 (29.0)	50 (3.9)
– ASD	1,130 (25.3)	40 (3.5)
– PDA	486 (10.9)	22 (4.5)
– Pulmonary Valve Disease	385 (8.6)	20 (5.2)
– CoA	174 (3.9)	11 (6.3)
– Aortic Valve Disease	128 (2.9)	6 (4.7)
– Mitral Valve Disease	104 (2.3)	<5 (.)
– Simple miscellaneous	14 (0.3)	<5 (.)

HLHS, hypoplastic left heart syndrome; I/HAA, interrupted/hypoplastic aortic arch; TGA, transposition of the great arteries; AVSD, atrioventricular septal defect; TAPVD, total anomalous pulmonary venous drainage; PA, pulmonary atresia; TOF, tetralogy of fallot; VSD, ventricular septal defect; CoA, coarctation of the aorta; ASD, atrial septal defect, PDA, patent ductus arteriosus.

Complex miscellaneous: vascular ring of aorta, pulmonary artery atresia, congenital malformation of heart chambers of unknown specification, congenital coronary artery aneurism, congenital portal vein anomaly, scimitar syndrome, congenital anomalies of the great veins, congenital aortopulmonary septal defect, Eisenmenger defect, see Supplemental Material for ICD-10 codes.

Simple miscellaneous: cor triatriatum, congenital vena cava stenosis, *other* congenital heart disease, see Supplemental Material for ICD-10 codes.

Note for *n* < 5, European personal data protection rules. GDPR: https://eur-lex.europa.eu/legal-content/EN/TXT/PDF/?uri=CELEX:32016R0679). GDPR inhibits the reporting of data on less than five individuals. GDPR took effect on May 25, 2018.

### Mortality

Overall, 71 deaths were registered in our cohort, corresponding to 1.6% of the total cohort of 4,469 children with CHD. The number of deaths and confounding and mediating variables for the overall cohort of children with CHD, those with simple- and those with complex CHD are depicted in Table 3.

**Table 3:** Mortalities and covariates in subgroups of CHD and NT.

	All NT values	NT ≥ 95th Centile	NT < 95th Centile	*p*
*All CHD, n (%)*	4,469 (100)	216 (4.8)	4,253 (95.2)	
– Mortalities, *n* (%)	70 (1.6)	8 (3.7)	62 (1.5)	0.01
– Mortality rate (%)				0.01
– 30 days	0.7	0.9	0.6	
– 3 months	0.9	1.4	0.9	
– 1 year	1.3	2.8	1.2	
– 5 year	1.6	3.6	1.4	
– PE, *n* (%)	255 (5.7)	18 (8.3)	237 (5.6)	0.09
– SGA, *n* (%)	464 (10.4)	16 (7.4)	448 (10.5)	0.14
– PTB < 37, *n* (%)	816 (18.3)	36 (16.7)	780 (18.3)	0.53
– PTB < 34, *n* (%)	432 (9.7)	18 (8.3)	414 (9.7)	0.50
– Extracardiac anomaly	652 (14.6)	49 (22.7)	603 (14.2)	<0.01
– Follow-up time (years), median (iqi)	6.4 (3.8;9.1)	5.3 (3.2;8.5)	6.4(3.9;9.1)	<0.01
*Simple CHD, n (%)*	3,715 (83.1)	154 (71.3)	3,561 (83.7)	<0.01
– Mortalities, *n* (%)*		<5 (.)	22 (0.6)	<0.01
– Mortality rate (%)				<0.01
– 30 days	0.2	0.7	0.2	
– 3 months	0.3	0.7	0.3	
– 1 year	0.5	1.3	0.5	
– 5 year	0.7	2.4	0.6	
– PE, *n* (%)	221 (5.9)	13 (8.4)	208 (5.8)	0.18
– SGA, *n* (%)	386 (10.4)	1 (7.1)	375 (10.5)	0.18
– PTB < 37, *n* (%)	701 (18.9)	27 (17.5)	674 (18.9)	0.66
– PTB < 34, *n* (%)	387 (10.4)	15 (9.7)	372 (10.4)	0.78
– Extracardiac anomaly	535 (14.4)	35 (22.7)	500 (14.0)	<0.01
– Follow-up time (years), median (iqi)	6.5 (3.9;9.1)	5.0 (3.1;8.1)	6.5 (3.9;9.2)	<0.01
*Complex CHD, n (%)*	754 (16.9)	62 (28.7)	692 (16.3)	<0.01
– Mortalities, *n* (%)*		<5 (.)	40 (5.8)	0.83
– Mortality rate (%)				0.83
– 30 days	2.9	3.2	2.9	
– 3 months	4.0	3.2	4.1	
– 1 year	5.2	6.5	5.1	
– 5 year	5.7	6.5	5.7	
– PE, *n* (%)	34 (4.5)	5 (8.1)	29 (4.2)	0.16
– SGA, *n* (%)	78 (10.3)	5 (8.1)	73 (10.5)	0.54
– PTB < 37, *n* (%)	115 (15.3)	9 (14.5)	106 (15.3)	0.87
– PTB < 34, *n* (%)*		<5 (.)	42 (6.1)	0.70
– Extracardiac anomaly	117 (15.5)	14 (22.6)	103 (14.9)	0.11
– Follow-up time (years), median (iqi)	6.0 (3.7;8.6)	6.3 (3.1;9.2)	6.0 (3.7;8.6)	0.98

PE, preeclampsia; SGA, small for gestational age; PTB < 37, preterm birth before 37 weeks of gestation; PTB < 34, preterm birth before 34 weeks of gestation; iqi, interquartile interval.

*European personal data protection rules. GDPR: (https://eur-lex.europa.eu/legal-content/EN/TXT/PDF/?uri=CELEX:32016R0679). GDPR inhibits the reporting of data on less than five individuals. GDPR took effect on May 25, 2018.

The number of deceased patients was higher in the group with a NT ≥ 95th centile (3.7%) vs. those with a NT < 95th centile (1.5%, *p* = 0.01, Table 3).

**Figure 2:**
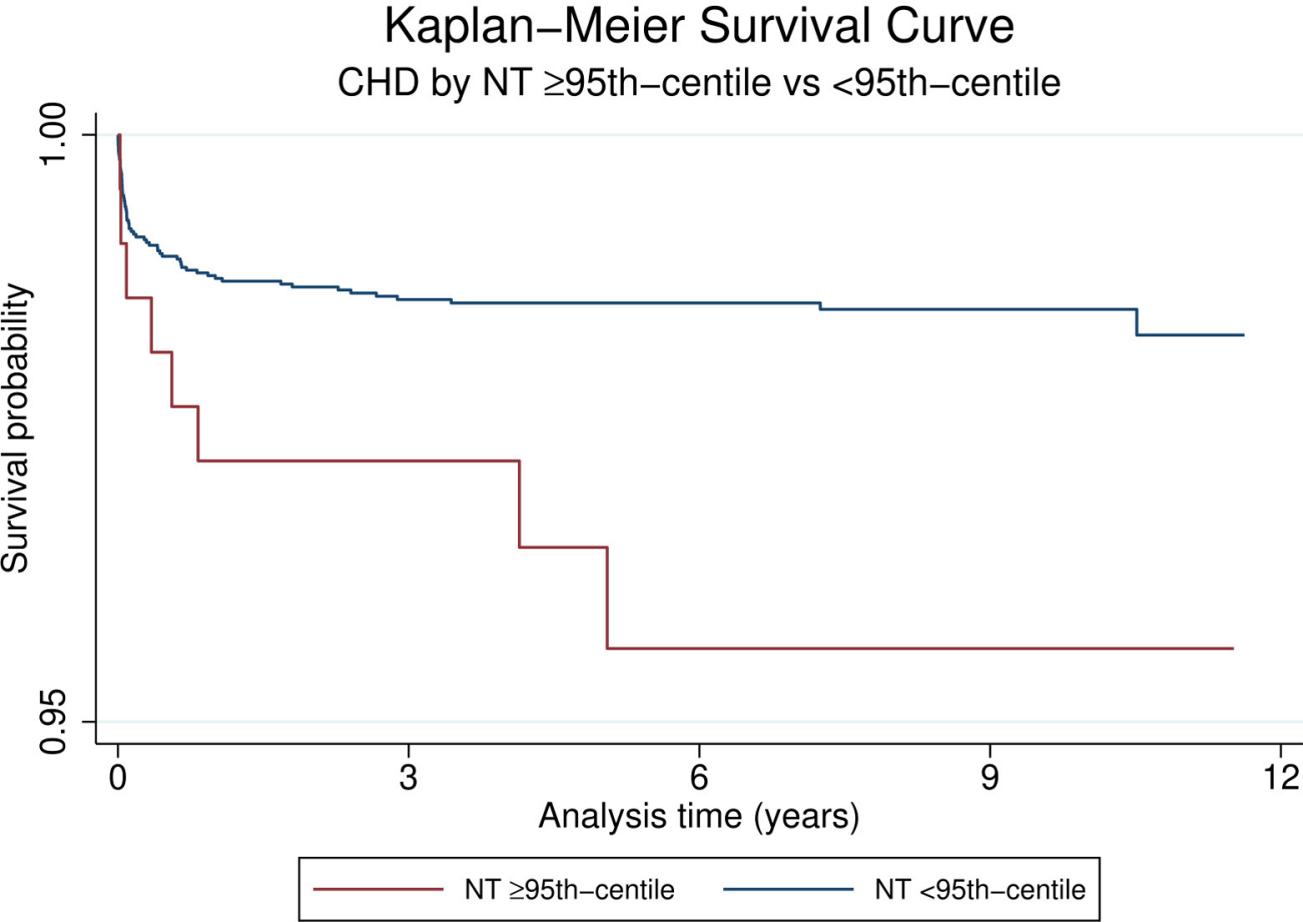
Kaplan-Meier survival curves of CHD by NT ≥ 95th centile vs. <95th Centile. *Note scaled y-axis to highlight region of difference of Kaplan-Meier curves*.

**Figure 3:**
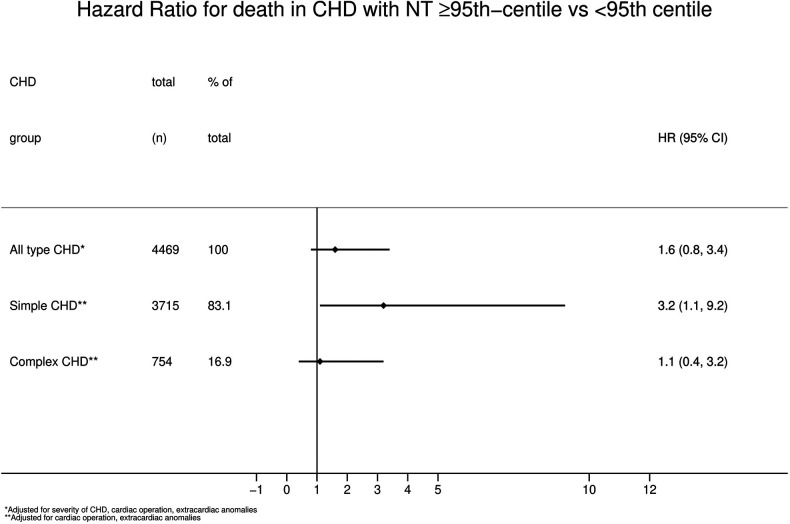
Hazard ratio for mortality in CHD with NT ≥ 95th centile vs. < 95th Centile. CHD, congenital heart defect; NT, nuchal translucency.

Looking at the overall group of children with CHD the HR for mortality was 2.6 (95% CI 1.3;5.4, *p* = 0.01, Table 3) when comparing children with a NT ≥ 95th centile vs. those with a NT < 95th centile. This is also demonstrated by the Kaplan-Meier survival estimates for NT ≥ 95th centile vs. <95th centile for all patients with CHD (Figure 2, log-rank-test *p* < 0.01). When considering a possible confounding effect of the severity or type of CHD (complex or simple CHD) and adjusting for this, this revealed a decline in HR to 1.8 (95%CI 0.9;3.8, *p* = 0.1). Further adjustment for confounding by cardiac operation and extracardiac anomaly only decreased the HR slightly to 1.6 (95%CI 0.8;3.4, *p* = 0.2, Figure 3). The initial fall in HR suggests confounding by the severity or type of CHD (complex or simple CHD), and further analysis in stratified groups of either complex or simple CHD was done.

Simple CHD had a significantly higher mortality rate and a HR of 3.2 (95% CI 1.1;9.2, *p* = 0.03, Figure 3) when having a NT ≥ 95th centile than a NT < 95th centile, adjusted for confounding by extracardiac anomalies and cardiac operation.

In contrast to simple CHD, complex CHD had no differences in mortality rate between a NT ≥ 95th centile and a NT < 95th centile (HR 1.1, 95%CI 0.4;3.2, *p* = 0.8, Figure 3), adjusted for extracardiac anomalies and cardiac operation.

The mean NT thickness among the deceased patients were 1.8 mm (sd = 0.6) for all CHD combined (mean 1.7 mm (sd = 0.4) vs. 3.0 mm (sd = 0.5) when NT < 95th vs. NT ≥ 95th centile), 1.9 mm (sd = 0.7) for simple CHD (mean 1.6 mm (sd = 0.4) vs. 3.1 mm (sd = 0.6) when NT < 95th vs. NT ≥ 95th centile) and 1.8 mm (sd = 0.6) for complex CHD (mean 1.7 mm (sd = 0.4) vs. 3.0 mm (sd = 0.6) when NT < 95th vs. NT ≥ 95th centile).

Mortality rates at 30-days, 3-months, 1-year and 5-years were higher and rose over time among those with a NT ≥ 95th centile, except for those with complex CHD (Table 3). For overall CHD and simple CHD the 5-year mortality rate in overall CHD was 3.6% for those with NT ≥ 95th centile and 1.4% for those with NT < 95th centile (*p* < 0.01, Table 3), and for simple CHD 2.4% vs. 0.6% (*p* < 0.01, Table 3).

The prevalence of PE, PTB and SGA did not differ between a NT ≥ 95th centile and a NT < 95th centile, but significantly more children had an extracardiac anomaly in the group with the larger NTs.

Adjusting for mediating effects of PE, PTB and SGA did not change the HR and significance in the above analysis. Adjusting for confounding effects of undergoing cardiac surgery did not change the HRs or significance in any of the above analysis. Adjusting for confounding by extracardiac anomalies only influenced the HR of mortality in the subgroup of simple CHD. It was not possible to analyze mortality rates and HRs for death for each single subtype of CHD due to the low numbers of deaths, limiting the basis for statistical comparison.

## Discussion

In this nationwide study on all liveborn children in Denmark from 2008 to 2018 with CHD, we found a significantly increased risk of mortality (HR 3.2) if children with simple CHD had a large NT (NT ≥ 95th centile) in first trimester compared to those with a NT < 95th centile.

Focus has previously been on the association between the NT and the presence of complex CHD, as well as the adverse outcomes for the children with an increased NT (12–14). No studies have focused on the NT as a possible predictor of excess mortality in children with CHD.

We know that children with complex CHDs are the most vulnerable. Interestingly, we found a correlation between an increased NT and mortality in children with *simple* CHD. This surprising finding has previously been described for outcomes such as long-term mortality in patients with atrial- and ventricular septal defects (45, 46), and neurodevelopmental disorders when exposed to preeclampsia in fetal life (35). It is important to note that these correlations do not imply causality, but likely point to a common cause among those with simple CHD—perhaps a genetic as we discuss below.

The reason for this paradox of vulnerability where the complex CHDs seems less affected by an increased NT than the simple, is most likely due to a small sample size of complex CHDs with NT ≥ 95 centile and the scarcity of the outcome of mortality. Another reason could be that an increased NT contributes with no measurable extra risk for the child with a complex CHD.

A “live birth bias” might also play a role (35). This type of survival bias could occur as pregnancies with a fetus with a complex CHD and a large NT are likely to be terminated or die *in utero*. A substantial part of the fetuses in Denmark with a prenatally diagnosed complex CHD are terminated (47). They would therefore not be represented in our cohort, be at risk, and add to the mortality in their group. In contrast, those with a simple CHD and a thick NT are born and add to the risk for said outcome.

The risk of a child dying from a simple CHD in Denmark is very low (30). In our study those with a simple CHD and an increased NT have a higher mortality, indicating that these children die from some other cause than their CHD. Unfortunately, we do not have data on the exact cause of death as we discuss in the limitations. With the close linkage of an increased NT to genetic abnormalities it is possible that abnormal genetics may in fact be the driver of a higher mortality. Although we excluded children with chromosomal abnormalities from our study, we did not have data on monogenic abnormalities, copy-number-variants or RASopathies (i.e., Noonan syndrome). These undetected abnormalities could cause an increased NT and an increased risk of mortality and this could explain why the simple CHDs with NT ≥ 95th centile have a higher mortality.

The nuchal translucency is lymphatic in its structure, and if increased thought to be due to cardiac or lymphatic problems (4, 15, 16). The lymphatic and cardiovascular system are developed around the same time in gestation and both systems are likely influenced by the same developmental disruptions (17, 48).

The entwinement of these systems are exemplified by the presence of lymphatic abnormalities in children with univentricular hearts (19, 49). Not being able to draw causal conclusion we can speculate that genetic factors, undetectable in this study, results in the combination of a challenged lymphatic system, evident by an increased NT, and a CHD, leading to increased risk of adverse outcomes.

### Strengths and limitations

Our study is based on a unique large national cohort with almost complete follow up and information collected from the first trimester screening scan, throughout the pregnancy and childhood for children with CHD.

Our cohort is comparable to previous studies on children with CHD, where higher prevalence of impaired maternal-fetal environmental factors such as preeclampsia, pre-term birth, low gestational age and low birthweight are seen compared to the general population (28, 41, 50–53). The mortality rate of children with CHD is comparable to previous studies on the Danish population with CHD (30).

We excluded children with chromosomal abnormalities from our study population based on data from the Danish Cytogenetic Central Registry. A limitation to our study is the unwanted inclusion of children with unidentified abnormal genetics that might bias our associations. As touched upon the presence of undetected copy-number-variants or RASopathies could be a common cause of an increased NT, CHD and higher mortality, explaining the higher mortality among those with NT ≥ 95th centile. This limits us from describing a direct association between increased NT and mortality. We do not have data on how many other genetic abnormalities of greater or lesser importance are present in the cohort. The effect of this bias has been argued to be small as such abnormalities are rare and as they effect will likely dilute with the size of the groups studied (32).

Another limitation is the use of several registers, which increases dependency on the validity of each register and the data within this. Contrary, we could not create this large a cohort without the registers, and the Danish National Patient Register is generally considered to have a high validity (54).

A limitation to our study is the scarcity of children with CHD and a high NT (*n *= 216, Table 3), and death as well. This makes it difficult to make strong statistical associations. The scarcity might be due to the overall combined low incidence of having both a CHD and an increased NT, or that a large proportion of prenatally diagnosed CHDs, especially those with a complex CHD, and a NT ≥ 95th centile are terminated (47). Care should be taken when interpreting the results of mortality in simple CHD with increased NT as the number of deaths are very low. Future research on a larger dataset or with a longer period of study could provide data containing more deaths, and this would then provide the possibility of testing the correlation again.

Although we have included *all* children with CHD and a NT-scan in the period of study, the low number of deaths make it difficult to show possible differences for subgroups of CHD. It was not possible to divide the mortalities onto their specific CHD, as the GDPR rules of reporting data would be violated due to low number of mortalities. It was also necessary to pool the mortalities of subtypes of CHD into larger subtypes (i.e., simple and complex), to be able to statistically compare groups.

We do not have exact cause of death in our database. We excluded those dead from unnatural causes such as suicide or trauma. We cannot infer causality in this study but point to important associations. The driving factor between the association of the NT and higher mortality is unknown. It is for future studies to investigate the causes underlying this.

### Perspectives

It has been discussed that with new fetal screening tools such as circulating free DNA the ultrasonic measurement of NT might be replaced (55). Based on our results an antenatally increased NT should not create worry for the parents of the child or change the current guidelines regarding increased NT. But an increased NT should spike the attention of the clinician caring for the child with CHD as there might be undetected causes linked to the increased NT that can also lead to worse outcomes. Our study supports a renewed interest in measuring the NT in relation to CHD and postnatal outcomes and opens for further research on the causes of this.

## Conclusion

An increased NT ≥ 95th centile is correlated with higher mortality in children with simple CHD, but the underlying cause is unknown and undetected abnormal genetics might explain the correlation rather than the increased NT itself, hence further research is warranted.

## Data Availability

Datasets are not publicly available. Data was drawn from the Danish National Patient registers with permission from Regional Data Protection Agency (approval number: P-2020-183). Requests to access the datasets should be directed to rasmus.eskild.kristensen@regionh.dk.
