# Supplementary material for: Increased nuchal translucency in children with congenital heart defects and normal karyotype—is there a correlation with mortality?
Source: Front Pediatr. 2023 Feb 17;11:1104179. doi: 10.3389/fped.2023.1104179 (PMC9981958; doi:10.3389/fped.2023.1104179)
Supplement: Supplementary file 1 [file Table1.docx]

Supplementary Table a.: CHD subtypes and ICD-10 codes

| **Diagnosis** | **ICD-10** |
| --- | --- |
| *Complex CHD* |  |
| - UVH | DQ204, DQ226 |
| - HLHS | DQ234 |
| - Common Arterial Trunk | DQ200 |
| - I/HAA | DQ251A, DQ252, DQ254 |
| - TGA | DQ201-203, DQ203A, DQ205 |
| - AVSD | DQ212, DQ218B |
| - TAPVD | DQ262 |
| - PA | DQ220, DQ220A, |
| - TOF | DQ213, DQ213A, DQ218C |
| - Ebsteins Anomaly | DQ225 |
| - Tricuspid Valve Disease | DQ224, DQ228, DQ229 |
| - Complex Miscellaneous | DQ254I, DQ258, DQ259, DQ255, DQ20, DQ208, DQ209, DQ245, DQ265, DQ266, DQ268, DQ268B, DQ269, DQ206, DQ214, DQ218A, |
|  |  |
| *Simple CHD* |  |
| - VSD | DQ210, DQ21, DQ218, DQ219 |
| - CoA | DQ251 |
| - Aortic Valve Disease | DQ253, DQ244, DQ230, DQ231, DQ231A |
| - Pulmonary Valve Disease | DQ243, DQ221, DQ256, DQ222, DQ223, DQ257 |
| - Mitral Valve Disease | DQ23, DQ232, DQ233, DQ238, DQ239 |
| - ASD | DQ211, DQ263, DQ264 |
| - PDA | DQ250 |
| - Simple Miscellaneous | DQ248, DQ242, DQ260 |
| Abbreviations: UVH, univentricular heart; HLHS, hypoplastic left heart syndrome; I/HAA, interrupted/hypoplastic aortic arch; TGA, transposition of the great arteries; AVSD, atrioventricular septal defect; TAPVD, total anomalous pulmonary venous drainage; PA, pulmonary atresia; TOF, tetralogy of Fallot; VSD, ventricular septal defect; CoA, coarctation of the aorta; ASD, atrial septal defect, PDA, patent ductus arteriosus. | |
